# Phenotypic insights into *ADCY5*‐associated disease

**DOI:** 10.1002/mds.26598

**Published:** 2016-04-08

**Authors:** Florence C.F. Chang, Ana Westenberger, Russell C. Dale, Martin Smith, Hardev S. Pall, Belen Perez‐Dueñas, Padraic Grattan‐Smith, Robert A. Ouvrier, Neil Mahant, Bernadette C. Hanna, Matthew Hunter, John A. Lawson, Christoph Max, Rani Sachdev, Esther Meyer, Dennis Crimmins, Donald Pryor, John G.L. Morris, Alex Münchau, Detelina Grozeva, Keren J. Carss, Lucy Raymond, Manju A. Kurian, Christine Klein, Victor S.C. Fung

**Affiliations:** ^1^ Movement Disorders Unit, Department of NeurologyWestmead HospitalSydneyAustralia; ^2^ Institute of NeurogeneticsUniversity of LübeckLübeckGermany; ^3^ TY Nelson Department of Neurology and NeurosurgeryChildren's Hospital at WestmeadWestmeadAustralia; ^4^ University of SydneySydneyAustralia; ^5^ Department of NeurologyBirmingham Children's HospitalBirminghamUnited Kingdom; ^6^ Molecular Neurosciences, Developmental Neurosciences Program, Institute of Child HealthUniversity College LondonLondonUnited Kingdom; ^7^ Department of Child Neurology, Sant Joan de Déu HospitalUniversity of BarcelonaSpain; ^8^ Sydney Medical SchoolUniversity of SydneyAustralia; ^9^ Hunter GeneticsJohn Hunter HospitalNewcastleAustralia; ^10^ Genetics of Learning Disability ServiceNewcastleAustralia; ^11^ Sydney Children's Hospitals NetworkRandwickAustralia; ^12^ Department of Medical GeneticsSydney Children's HospitalRandwickAustralia; ^13^ Neurology DepartmentGosford HospitalGosfordAustralia; ^14^ Neurology DepartmentSt George HospitalKogarahAustralia; ^15^ Department of Medical Genetics, Cambridge Institute for Medical ResearchUniversity of CambridgeCambridgeUnited Kingdom; ^16^ Department of HaematologyUniversity of Cambridge, NHS Blood and Transplant CenterCambridgeUnited Kingdom; ^17^ Wellcome Trust Sanger InstituteHinxtonCambridgeUnited Kingdom; ^18^ College of Medical and Dental StudiesUniversity of BirminghamBirminghamUnited Kingdom

**Keywords:** adenylyl cyclase, dyskinesia, chorea, dystonia, cerebral palsy

## Abstract

**Background:**

Adenylyl cyclase 5 (*ADCY5*) mutations is associated with heterogenous syndromes: familial dyskinesia and facial myokymia; paroxysmal chorea and dystonia; autosomal‐dominant chorea and dystonia; and benign hereditary chorea. We provide detailed clinical data on 7 patients from six new kindreds with mutations in the *ADCY5* gene, in order to expand and define the phenotypic spectrum of *ADCY5* mutations.

**Methods:**

In 5 of the 7 patients, followed over a period of 9 to 32 years, *ADCY5* was sequenced by Sanger sequencing. The other 2 unrelated patients participated in studies for undiagnosed pediatric hyperkinetic movement disorders and underwent whole‐exome sequencing.

**Results:**

Five patients had the previously reported p.R418W *ADCY5* mutation; we also identified two novel mutations at p.R418G and p.R418Q. All patients presented with motor milestone delay, infantile‐onset action‐induced generalized choreoathetosis, dystonia, or myoclonus, with episodic exacerbations during drowsiness being a characteristic feature. Axial hypotonia, impaired upward saccades, and intellectual disability were variable features. The p.R418G and p.R418Q mutation patients had a milder phenotype. Six of seven patients had mild functional gain with clonazepam or clobazam. One patient had bilateral globus pallidal DBS at the age of 33 with marked reduction in dyskinesia, which resulted in mild functional improvement.

**Conclusion:**

We further delineate the clinical features of *ADCY5* gene mutations and illustrate its wide phenotypic expression. We describe mild improvement after treatment with clonazepam, clobazam, and bilateral pallidal DBS. *ADCY5*‐associated dyskinesia may be under‐recognized, and its diagnosis has important prognostic, genetic, and therapeutic implications. © 2016 The Authors. Movement Disorders published by Wiley Periodicals, Inc. on behalf of International Parkinson and Movement Disorder Society

In 2001, Fernandez and colleagues reported a single kindred with a novel clinical syndrome described as familial dyskinesia and facial myokymia (FDFM; OMIM#606703).^1^ Affected members presented with childhood‐ or adolescent‐onset distal choreiform movements with facial myokymia. In 2012, whole‐exome sequencing of 1 member of this original kindred revealed a c.2176G>A (p.A726T) mutation in the adenylyl cyclase 5 (*ADCY5*) gene. The pathogenicity of this change was supported by its absence in control cases and transmission in affected family members shown by cosegregation analysis.^2^ Subsequently, a novel c.1252C>T (p.R418W) *ADCY5* mutations in 2 sporadic cases of childhood‐onset paroxysmal chorea and dystonia was identified.^3^ Recently, 2 additional kindreds with autosomal‐dominant mode of inheritance (p.R418W and c.2088+1G>A leading to haploinsufficiency)^4^, ^5^ and 1 sporadic patient (p.R418W)^4^ have been reported with a syndrome of chorea and dystonia without paroxysmal episodes.^4^, ^5^

In 2011, at the 5th International Dystonia Symposium in Barcelona, we reported on 4 patients with a syndrome we described as “familial choreoathetosis with exacerbations during drowsiness”^6^ (Supporting Fig. 1). All were initially diagnosed with dyskinetic cerebral palsy, but based on their clinical features, we suspected they had an unique, probably genetically determined, syndrome. Here, we provide detailed descriptions of those patients and report that all 4 patients have now been shown to harbor an identical *ADCY5* gene mutation, as well as an additional sporadic case with the identical phenotype and mutation. Two further probands with milder, but overlapping, phenotypes had previously been diagnosed syndromically as autosomal‐dominant myoclonus‐dystonia and sporadic, infantile‐onset chorea without dystonia before the discovery of their novel *ADCY5* mutations. From these data and a review of the other 6 kindred reported in the literature, we describe detailed clinical findings and highlight the clinical spectrum of *ADCY5*‐related neurological disorders.

## Patients and Methods

### Patients and Genetic Analysis

All study participants gave written informed consent for genetic testing as well as for disclosure of results and videos. The study was approved by the local ethics committee and was carried out in accord with the Declaration of Helsinki.

**Figure 1:**
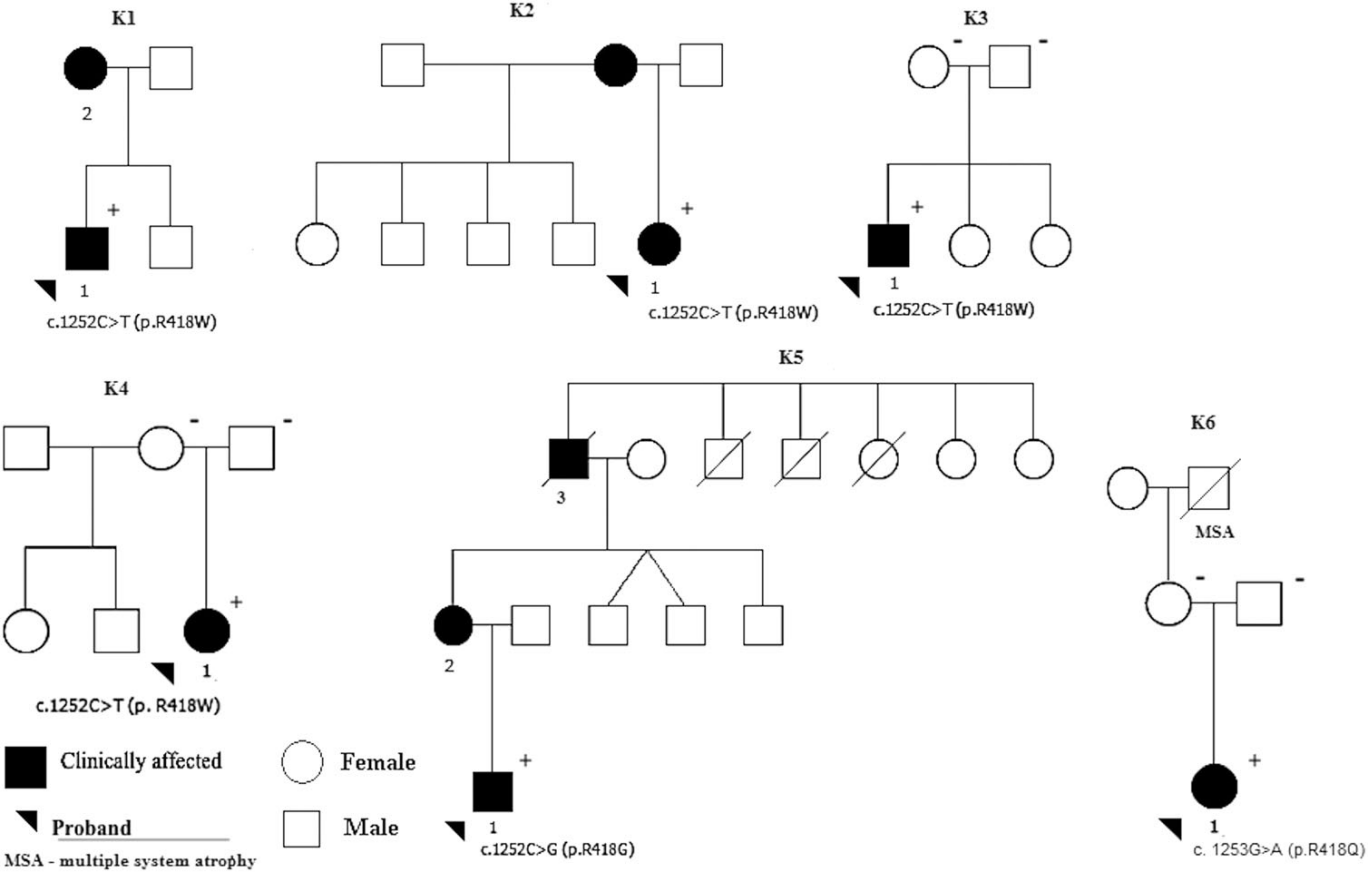
Kindred pedigrees. Arrowheads indicate probands of each pedigree; black symbols, affected; white symbols, unaffected members; slashed symbols, deceased individuals. In the genetically analyzed individuals, + denotes the presence of the *ADCY5* mutation, – denotes the absence of *ADCY5* mutation.

Four patients from 3 kindreds (K1, K2, and K5) and 1 sporadic patient (K3‐1) followed in one institution over a period of 9 to 32 years were suspected of harboring *ADCY5* mutations based on their clinical features. Thus, for subjects K1‐1, K2‐1, K3‐1, and K5‐1 (Fig. 1), as well as the clinically unaffected parents of K3‐1, Sanger sequencing of exons 2, 8, and 10 (harboring mutations reported in the literature) and corresponding exon‐intron junctions (a minimum of 50 base pairs of intronic DNA flanking each of the three analyzed exons) of the *ADCY5* gene was carried out using an ABI 3500XL automated sequencer (Applied Biosystems, Foster City, CA). In addition, we performed haplotype analysis in the 3 unrelated carriers of the p.R418W (cases K1‐1, K2‐1, and K3‐1) change by genotyping five short tandem repeat markers situated in close proximity (D3S3674, D2S3636, D3S1269, and D3S3573) or within (D3S1267) the *ADCY5* gene.

A further 2 unrelated patients (K4‐1 and K6‐1 and their clinically unaffected parents) who participated in a whole‐exome sequencing study of undiagnosed pediatric movement disorder cases were also identified as having *ADCY5* mutations and included in the clinical analysis (Supporting Methods).

Genetic analysis was targeted to genes causing childhood dyskinesia. Identified *ADCY5* variants were confirmed in cases K4‐1 and K6‐1 as well as its absence in their parents with direct Sanger sequencing of the relevant exon using standard techniques.

For the in silico prediction of pathogenicity/functional importance of the identified mutations we used: (1) MutationTaster^7^; (2) PolyPhen2^8^; (3) SIFT^9^; and (4) combined annotation‐dependent depletion (CADD)^10^ tools. In addition, we assessed the frequency of the detected changes in the Exome Aggregation Consortium (ExAC) Browser.^11^

### Literature Review

Previous case reports of *ADCY5* mutation were collected from published articles and its reference list, found on Pubmed using the following keywords: “adenylyl cyclase 5” and “adenylate cyclase 5.”

## Results

The kindred pedigrees and mutations are displayed in Figure 1. The clinical data from our patients as well as the 6 previously reported kindreds with *ADCY5* mutations are summarized in Table 1 and Supporting Table 1. Detailed history, examination findings, and investigations in our patients are available in the Supporting Materials (History and examination and Supporting Table 2). Our patients had a variable phenotype, but with overlapping clinical features. We will summarize the phenomenology, genetic findings, and genotype‐phenotype correlations.

**Table 1:** Clinical features and *ADCY5* mutations reported to date

		Frequency Range	
Clinical Features	No. of Cases (Where Reported)	% (n = 20)	% (Where Reported)	Our Series
Presentation				
Birth to 6 months	4/19	20	21	3/7
7 months to 2 years	7/19	35	37	3/7
>2 years	8/19	40	42	1/7
Syndrome				
CWEDD	5/20	25	25	5/7
MD	1/20	5	5	1/7
IOIC	1/20	5	5	1/7
COPCD	2/20	10	10	0/7
FDFM	6/20	30	30	0/7
EOADCD	2/20	10	10	0/7
BHC	3/20	15	15	0/7
Gene mutation				
c.1252C>T	10/20	50	50	5/7
c.1252C>G	1/20	5	5	1/7
c.1253G>A	1/20	5	5	1/7
c.2176G>A	6/20	30	30	0/7
c.2088+1G>A	2/20	10	10	0/7
Phenomenology				
Chorea	18/19	90	95	6/7
Facial dyskinesia	11/20	55	55	5/7
Axial hypotonia	8/16	40	50	6/7
Dystonia	14/17	70	82	6/7
Myoclonus	3/11	15	27	2/7
Spasticity	6/20	30	30	3/7
Intellectual disability	2/20	10	10	2/7
upward gaze palsy	7/13	35	54	4/7
Motor regression	6/20	30	30	2/7
Epilepsy	1/19	5	5	0/7
Duration of episodic exacerbation				
Minutes to hours	8/10	40	80	6/7
Hours to days	1/10	5	10	0/7
Constant	1/10	5	10	1/7
Exacerbating factors				
Action	8/16	40	50	6/7
Stress	10/16	50	63	4/7
Awakening	3/16	15	19	0/7
Drowsiness	7/16	35	44	6/7
Improvement with				
Clonazepam	4/15	20	27	4/7
Clobazem	2/15	10	13	2/7
Carbamazepine	1/15	5	7	1/7
Propranolol	1/15	5	7	NT
Acetazolamide	2/15	10	13	NT
Trihexyphenidyl	3/15	15	20	0/7
Tetrabenazine	2/15	10	13	NT
Caffeine	1/15	5	7	1/7
Action	1/15	5	7	0/7

NT, not tried; CWEDD, choreoathetosis with exacerbation during drowsiness; MD, myoclonus dystonia; COPCD, childhood onset paroxysmal choreiform and dystonic movements; FDFM, Familial dyskinesia and facial myokymia; EOADCD, early onset autosomal dominant chorea and dystonia; IOIC, infantile onset isolated chorea; BHC, benign hereditary chorea

### Phenomenology

#### Dyskinesias

The dominant feature in our patients is the infantile‐ or early childhood‐onset dyskinesia. The movements were initially episodic in all our subjects occurring for seconds to hours in duration. Although exacerbating factors could often be identified, they lacked the stereotyped trigger and duration of many paroxysmal movement disorders, leading us to prefer the term episodic rather than paroxysmal. The dyskinesias were similar in all subjects, being characterized by brief jerks and twitches that were commonly classified as chorea, ballism, or choreoathetosis. The label choreoathetosis was used when there were slower writhing movements,^12^ usually in the upper limbs, in addition to the superimposed choreiform movements. They were worse with mental activity, anxiety, and prominent during action. Often, classification of the phenomenology was uncertain, even by movement disorder specialists. Although classified as predominantly myoclonus only in patient K5‐1 (who also had some slower, choreiform movements), most of the other patients in our series also had their dyskinesias labeled as myoclonus by some clinicians, emphasizing their twitchy or jerky character. The distribution of the dyskinesia was generalized, and 4 of 7 had facial dyskinesia. In 3 of 7 patients, the dyskinesias became more continuous or constant with increasing age, but involuntary movements could still be absent at rest. The dyskinesias are illustrated in the video files that accompany this article (see Video 1, Segment 1; Video 2, Segment 2; and Video 3, Segments 2 and 3).

A notable characteristic was that all of our subjects reported exacerbations of dyskinesia that interrupted sleep. Four of seven subjects had a history of dyskinesias during drowsiness that prevented sleep initiation, sometimes writhing for hours after retiring to bed, whereas the other subjects reported dyskinesias associated with awakening in early morning after sleep. Subject K3‐1 had a sleep study at age 26 and it showed poor sleep efficiency (32%) because of long sleep latency associated with the presence of dyskinesias. There was also mild hypopnea preceding involuntary movements of the upper limb followed by trunk, then hip and knee flexion movements mostly on sleep arousal and once during stage 2 sleep. These movements lasted for up to 30 seconds, too long for periodic limb movements and with no evidence of epileptiform EEG activity (Supporting Fig. 3). After commencement of clonazepam (0.01‐0.20 mg/kg) or clobazam at (0.2 mg/kg), 4 and 2 of 7 of our subjects, respectively, reported improvement in dyskinesia that previously disrupted sleep. Whereas most subjects reported no benefit with carbamazepine and worsening of episodic dyskinesia with caffeine, 1 subject reported benefit with caffeine and another with carbamazepine. Treatment with levodopa, sodium valproate, tetrabenazine, trihexyphenidyl, pregabalin, gabapentin, phenobarbitone, or baclofen were inevitably ineffective. Bilateral pallidal DBS was performed in subject K2‐1 and resulted in improvement in her choreoathetosis. In 2 of 7 subjects, episodic dyskinesia worsened in early adulthood associated with motor regression and loss of ambulation. Two of the relative milder affected subjects (K5‐1 and K6‐1) had an increase in dyskinesia during childhood that caused falls without motor regression or loss of ambulation (see Video 3, Segment 4). Surface electromyography (sEMG) study was available for 2 subjects. In subject K3‐1, upper limb sEMG study revealed action‐induced choreoathetosis (Supporting Fig. 4). In subject K5‐1, at age 16, sEMG of the upper limbs revealed frequent 100‐ to 250‐ms bursts of muscle activity with sustained bursts between 500 and 2,500 ms during nose‐targeted position associated with cocontraction of antagonistic muscle groups, consistent with the combination of myoclonus and dystonia^13^, ^14^, ^15^ (Supporting Fig. 5).

### Dystonia

Axial hypotonia was prominent in 6 subjects, caused significant functional impairment, and was associated with a more severe phenotype. Infantile axial hypotonia with motor milestone delay was the presenting feature in 5 of 7 of our subjects. This led to a distinctive “frog‐like” method of ambulation observed in 2 of our subjects during childhood (see Video 1, Segment 3; Video 2, Segment 1). Interestingly, clonazepam significantly reduced axial hypotonia in 2 of 6 subjects, leading to an improvement in ambulation (see Video 2, Segment 3) and stability during sitting. Despite the choreoathetosis improving in subject K2‐1 after pallidal DBS, axial dystonia did not improve, leading to limited improvement in functional ability.

In 6 of 7 subjects, action‐induced dystonic posturing was observed in addition to their chorea, leading to the labeling of the movements as choreoathetosis. Generalized dystonic spasms were observed separate to their episodic dyskinesia in 3 subjects. These dystonic spasms consisted of truncal extension, retrocollis, and upper limb extension lasting for seconds (see Video 3, Segment 3), but which could recur in clusters lasting hours, especially during intercurrent illness. One subject had short dystonic spasms provoked by laughter and sneezing, and another subject experienced longer duration dystonic spasms with intercurrent illness or high ambient temperature.

#### Gait

Six of seven subjects had delayed motor milestones. Sitting was achieved between age 1.5 years to 7 attributed to axial hypotonia, and only 2 subjects with a milder phenotype could walk independently by the age of 3. All other subjects required gait assistance and achieved this between age 2.5 years and 13. Their gait impairment is secondary to axial hypotonia and action‐induced episodic dyskinesia. Without a walker, 2 subjects had distinctive “frog‐like” gait—sitting or lying prone with the legs crossed, then uncrossing them to propel the body forward while using their arms to commando crawl.

In early adulthood, 2 subjects lost the ability to walk with support secondary to axial hypotonia and increased episodic dyskinesia. One subject temporarily had reduced axial hypotonia and dyskinesia on a combination of clonazepam and l‐dopa and regained the ability to walk for a few years. Subject K5‐1 had falls secondary to truncal extension dystonic spasms and this improved after clonazepam.

#### Cognitive Development

Five subjects had delayed language development, speaking single words between age 2 and 7. Speech delay was associated with the presence of facial dyskinesia, which was present in 4 of these 5. However, 3 subjects subsequently acquired normal language ability and cognition. Two subjects had intellectual disability, and neuropsychological testing revealed mild‐to‐moderate impairment in learning, reasoning, and complex problem solving ability. Three of five subjects with a severe motor phenotype nevertheless had normal cognitive function. Both of the milder affected patients (K5‐1 and K6‐1) had normal cognitive function.

#### Eye Movements

Abnormal saccades were present in 5 of 6 patients where eye movements were documented. Four had absent saccadic upgaze (with 1 also initiating horizontal saccades with head thrust), whereas subject K4‐1 had prolonged vertical saccadic latencies. Only 1 subject had normal vertical saccadic eye movements. All subjects had normal pursuit. One subject (K6‐1) did not have detailed eye movement examination.

#### Spasticity

Three of seven had lower limb spasticity on examination with increased tone, hyper‐reflexia, and extensor plantar responses.

### Genetic Studies

Each of the probands in kindreds K1 to K4 carried a previously reported *ADCY5* c.1252C>T (p.R418W) mutation.^3^, ^5^ The mutation was absent in the parents of K3‐1 and K4‐1. None of the other family members of K1 and K2 (including the clinically affected parents) were available for genetic testing. Genotyping analysis performed in the probands of K1, K2, and K3 revealed no shared haplotype.

Cases K5‐1 and K6‐1 had novel heterozygous missense changes (c.1252C>G; p. R418G and c.1253G>A; p.R418Q, respectively). Both of the novel variants were not present in >60,000 exomes from the ExAC Browser, and the amino acid they affect is highly conserved throughout different species (Supporting Fig. 2). In addition, they were predicted to be pathogenic by three different prediction tools and their functional importance is indicated by very high CADD scores (Supporting Table 3). Mutations in other genes causing childhood‐onset dyskinesia were excluded in patient K6‐1 (Supporting Table 2).

### Phenotype‐Genotype Correlations

Given the relatively small number of families, definitive conclusions about phenotype‐genotype correlation are not possible. However, it was notable that all 4 kindreds presenting with the more severe phenotype, in terms of dyskinesias, axial hypotonia, and motor developmental delay, had the *ADCY5* p.R418W mutation. The other 2 probands who presented with milder phenotype had *ADCY5* p.R418G and p.R418Q mutations, respectively.

## Discussion

To date, 6 kindreds and 15 sporadic patients with *ADCY5* gene mutations have been reported in the literature.^1^, ^3^, ^4^, ^5^, ^16^ In the present study, we report novel clinical and genetic findings in 7 new cases (4 belonging to three unrelated families and 3 sporadic cases). Our findings are of significance given that, in addition to reporting two novel mutations, we expand and more clearly delineate the phenotypic spectrum of *ADCY5*‐related disease.

### Clinical Spectrum of *ADCY5* Mutations

We have shown that patients with various *ADCY5* gene mutations can present with a wider variety of movement disorder syndromes, similar to what was previously reported.^16^, ^17^ We describe novel clinical features associated with *ADCY5*‐related dyskinesia that distinguish this disorder from other hyperkinetic movement disorders. The frog‐like gait in childhood and worsening of episodic choreoathetosis during drowsiness are distinct features not observed in other hyperkinetic movements disorders.

The importance of intermittent exacerbations of choreoathetosis in *ADCY5* gene mutation carriers was first suggested in the report of familial dyskinesia and facial myokymia kindred in 2001^1^ (although the latter disproven by subsequent neurophysiological studies as facial chorea^17^) and in reports of “paroxysmal” chorea in 2014.^3^ We prefer to use the term “episodic” exacerbations because of the variable duration of the exacerbations and lack of precise triggers observed in *ADCY5* gene mutation patients, as opposed to sudden self‐limited episodes in response to consistent triggers that characterizes paroxysmal dystonia^18^ or paroxysmal dyskinesia.^19^ The duration of the episodic worsening of choreoathetosis lasts minutes to hours in our patients, and even up to days during intercurrent illness. A further important point differentiating *ADCY5* gene mutations from other classical causes of paroxysmal dyskinesia is the presence of a normal neurological examination between episodes in the latter,^20^ with the exception of *GLUT1* mutations.^21^ Last, the majority of movement disorders improve during sleep,^22^, ^23^ with the exception of familial paroxysmal hypnogenic dystonia, usually a manifestation of nocturnal frontal lobe epilepsy, which mostly occurs out of, rather than at the onset or initiation of, sleep.^22^, ^24^ Nevertheless, in all of our subjects, the nocturnal exacerbations led to the suspicion of a seizure disorder and investigation with video‐EEG telemetry, which was invariably negative. Our study is the first to further delineate the relationship between dyskinesia exacerbation and sleep. Previous studies reported worsening of paroxysmal dyskinesia during sleep,^3^, ^16^, ^25^ but our study is the first to provide formal polysomnographic data on patients with *ADCY5* mutation. Our patients display marked exacerbation during both drowsiness, associated with a prolonged sleep latency, and during sleep arousal. We suggest that exacerbations of choreoathetosis during drowsiness with prolonged sleep latency are a major clue to the presence of *ADCY5* mutations. An explanation for dyskinesia worsening during sleep could be the distribution of adenylyl cyclase 5 in the brain and its function. Adenylyl cyclase 5 is the major isoform of adenylyl cyclase in the nucleus accumbens.^26^ In animal studies, neuronal firing frequency in the nucleus accumbens has been shown to control the level of cortical arousal during the sleep‐wake cycle and also pharmacologically stimulated motor activity.^27^, ^28^, ^29^ Therefore, a gain of function in adenylyl cyclase 5 could potentially lead to increased arousal during sleep and increased motor activity in the form of dyskinesia. The frequent sleep arousal from gain of function could be the cause of the long sleep latency and exacerbation of dyskinesia, rather than dyskinesia preventing a restful sleep. Our study also reports symptomatic benefit with clonazepam or clobazam.^16^ Clonazepam and clobazam have an indirect inhibitory effect on adenylyl cyclase 5 activity,^30^, ^31^, ^32^ potentially counteracting the gain of function that has been shown to occur with the p.R418W mutation.

Our study is also the first to report marked improvement of choreoathetosis, albeit with only mild functional improvement, after bilateral globus pallidus DBS. The subject acquired independence in feeding and usage of the communication board. The lack of improvement in the dystonia severity scale can be explained by the limited effect of DBS on her axial hypotonia.

Most of the patients in our series were initially diagnosed with dyskinetic cerebral palsy, raising the possibility that *ADCY5*‐associated dyskinesia may be under‐recognized. Greater recognition of the distinctive clinical features that we and others^2^, ^6^, ^17^ have described should prompt testing for *ADCY5* gene mutation in patients with infantile‐ or childhood‐onset hyperkinetic disorder. The diagnosis of an *ADCY5* gene mutation has potential prognostic, genetic counseling, and therapeutic implications.

Family K5 was given a syndromic diagnosis of myoclonus‐dystonia, but tested negative for mutations the epsilon sarcoglycan gene (*SGCE*).^33^ On review of the phenomenology (see Video 3, Segment 2), although some of the involuntary movements could be labeled as chorea, there are also other movements that are shock‐like, occurring in the distal and proximal upper limbs, face, and trunk. Phenotypic overlap between myoclonus‐dystonia and benign hereditary chorea has previously been acknowledged.^34^, ^35^ When myoclonus and dystonia coexist, it can also be difficult to differentiate from chorea neurophysiologically, given that they both produce brief phasic, as well as tonic, sEMG activity.^36^ Interestingly, early‐onset myoclonus and dystonia, especially axial dystonia, have been reported as being markers of *SGCE* mutation‐positive versus *SGCE* mutation‐negative myoclonus‐dystonia patients.^37^ Our proband K5‐1 had axial hypotonia (as observed in 4 of our other *ADCY5* kindreds), rather than the more typical axial hypertonia that is reported in *SGCE*‐positive myoclonus‐dystonia. Therefore, we suggest that *ADCY5* gene mutations should be considered in the differential diagnosis of patients diagnosed with *SGCE*‐negative myoclonus‐dystonia, especially if there is axial hypotonia rather than hypertonia. The presence of prominent facial dyskinesia may also be an important clue, given that facial myoclonus is usually absent in SGCE‐positive myoclonus‐dystonia.^37^

Three of seven of our subjects had involuntary movements during infancy (K4‐1, K5‐1, and K6‐1). Infantile onset chorea has a wide differential diagnosis, both acquired and hereditary; however chorea almost always disappears during sleep,^38^ in contrast to our subject who had episodic exacerbations of chorea during arousal from sleep. Contrasting our patient's presentation with other acquired and hereditary causes of childhood‐onset chorea, Sydenham chorea tends to occur in childhood rather than in infancy and causes continuous rather than episodic generalized chorea, often associated with neuropsychiatric symptoms.^39^ Neuroimaging abnormalities, history of birth injury, and a nonprogressive course helps to differentiate dyskinetic cerebral pallsy from *ADCY5* mutations. Benign hereditary chorea is an autosomal‐dominant, nonprogressive infantile or childhood‐onset condition presenting with generalized chorea that usually diminishes or disappears with age,^40^ differing from *ADCY5* mutations in which choreoathetosis is often initially episodic and progressively becomes constant.^1^, ^3^ Therefore, we propose that infantile‐onset chorea, with exacerbation during drowsiness or arousal, should alert the clinician to the possibility of an *ADCY5* mutation.

The majority of our subjects had normal cognitive development despite subjects with initial language milestone delay. Two subjects had mild‐to‐moderate intellectual disability that was nonprogressive. The lack of cognitive decline differentiates *ADCY5* mutation from neurodegenerative conditions such as Huntington's disease, which presents with chorea and dementia.

### Mutational Spectrum of *ADCY5‐*Related Disease

The *ADCY5* p.R418W mutation present in 5 of our subjects has previously been reported and its pathogenicity established. Here, we also report two novel mutations resulting in p.R418G and p.R418Q changes, which affect the same amino acid in the adenylyl cyclase protein that is highly conserved across species. Our in silico analysis and database search, as well as the phylogenetic conservation^3^ (Supporting Fig. 2), of the affected amino acid strongly suggest that both of the novel mutations we detected are likely pathogenic. Furthermore, the *ADCY5* gene is one of the top 1% of genes intolerant to rare variants.^41^ The p.R418G and p.R418Q *ADCY5* mutations replace positively charged arginine with neutrally charged glycine and glutamine, respectively, and may result in tertiary structural and thus functional change to the protein product.

The adenylyl cyclase 5 pathway lies downstream from two known genetic causes of childhood‐ or adult‐onset dystonia. Mutations in the *Tor1A* gene are the most common genetic cause of childhood‐onset generalized dystonia. Cellular models suggest that the *Tor1A* gene mutation induces inhibition of the cyclic adenosine monophosphate response to an adenylyl cyclase 5 agonist.^42^ DYT25 causes adult‐onset generalized dystonia attributed to loss of function in the *GNAL* gene.^43^, ^44^ This gene codes for guanine nucleotide‐binding protein, alpha‐activating activity polypeptide, olfactory type [Ga(olf)], which is abundant in the striatum^45^). Ga(olf) protein activates adenylyl cyclase 5 linked to D1 receptors in the striatum.^46^ Thus, both genetic causes of dystonia converge on adenylyl cyclase 5, providing a potential link between previously known causes of dystonia and *ADCY5‐*associated neurological disease.

By analyzing parental DNA, we have shown that at least in our 3 sporadic patients (K3‐1, K4‐1, and K6‐1), pathogenic mutations arose de novo. Therefore, the absence of a positive family history does not exclude the possibility of an *ADCY5* mutation in individuals, and our data suggest that this codon may be especially susceptible to spontaneous mutations.

## Conclusion

In summary, our study further delineates the clinical features of *ADCY5* gene mutations and illustrates its wide phenotypic and varied phenomenological presentation. We describe, for the first time, functional improvement post‐treatment with clonazepam or clobazam and the potential usefulness of pallidal DBS in medication refractory cases. This novel condition may be under‐recognized, and its diagnosis has important prognostic, genetic counseling, and therapeutic implications for the patients and their families. We propose that the characteristic clinical findings that provide a clue to the diagnosis are generalized action‐induced choreoathetosis associated with episodic exacerbations during the early phase of sleep as well as axial hypotonia.

## Author Roles

(1) Research Project: A. Conception, B. Organization, C. Execution, (2) Statistical Analysis: A. Design, B. Execution, C. Review and Critique; (3) Manuscript: A. Writing of the First Draft, B. Review and Critique.

F.C.F.C.: 1A, 1B, 1C, 3A

A.W.: 1A, 1B, 1C, 3B

R.C.D.: 1A, 1B, 1C, 3B

M.S.: 3B

H.S.P.: 3B

B.P.‐D.: 3B

P.G.‐S.: 3B

R.A.O.: 3B

N.M.: 1A, 1B, 1C, 3B

B.C.H.: 1A, 1B, 1C, 3B

M.H.: 3B

J.A.L.: 3B

C.M.: 3B

R.S.: 3B

E.M.: 1A, 1B, 1C, 3B

D.C.: 3B

D.P.: 3B

J.G.L.M.: 3B

A.M.: 3B

D.G.: 3B

K.S.C.: 3B

L.R.: 3B

M.A.K.: 1A, 1B, 1C, 3B

C.K.: 1A, 1B, 1C, 3B

V.S.C.F.: 1A, 1B, 1C, 3B

## Financial Disclosures

F.C.F.C. is the recipient of the Neville‐Brown scholarship from University of Sydney. H.S.P. has received an unrestricted educational grant for meeting attendance from Medtronic and Britannia London. R.C.D. received an honorarium from Bristol‐Myers Squibb. V.S.C.F. receives a salary from NSW Health, has received research grants from the National Health and Medical Research Council of Australia and AbbVie, and is on advisory boards and/or has received travel grants from Abbott/AbbVie, Allergan, Boehringer Ingelheim, Hospira, Ipsen, Lundbeck, Merz, Novartis, Global Kinetics, Solvay, and UCB.

## Supporting information

Additional Supporting Information may be found in the online version of this article at the publisher's web‐site.

Supplementary Information 1

Supplementary Information 2

Supplementary Information Figure 1

Supplementary Information Figure 2

Supplementary Information Figure 3

Supplementary Information Figure 4

Supplementary Information Figure 5

Supplementary Information Table 1

Supplementary Information Table 2

Supplementary Information Table 3

Supplementary Information Video 1

Supplementary Information Video 2

Supplementary Information Video 3
