# Supplementary material for: Phenotypic insights into ADCY5‐associated disease
Source: Mov Disord. 2016 Apr 8;31(7):1033–40. doi: 10.1002/mds.26598 (PMC4950003; doi:10.1002/mds.26598)
Supplement: Supplementary file 1 — Supplementary Information 1 [file MDS-31-1033-s001.docx]

Supplementary Material

K1 Case 1

This twenty-year-old Caucasian man is the son of K1-2. He was born after a normal pregnancy and uneventful elective Caesarean delivery. He was noted to have motor developmental delay at seven months and generalized action-induced choreoathetosis from one year of age. He had delayed motor and language milestones. He could roll at age five years and sat independently at the age of seven years. He never learnt to crawl or walk independently but was able to use a walker at age one. He used single words at age seven and spoke in full sentences at the age of eight. There was normal intellectual development. The generalized episodic choreoathetosis began at age one and fluctuated in frequency. Episodes of choreoathetosis lasted for several minutes and occurred in clusters every fifteen minutes when most frequent. He could sleep for the first four hours without exacerbation of involuntary movements. However, these disturbed his sleep after midnight. The episodic movements were thought to be consistent with epilepsy and he had video EEG telemetry. Clonazepam 1.5mg daily seemed to reduce these movements. He was tried on levodopa (100mg daily for three months), valproate, clobazam and carbamazepine without benefit. The involuntary movements were worse during sleep after midnight, attempted action, emotional stress and intercurrent illness. There was no motor regression. Only his mother was affected in the family (K1-2).

On examination at age ten years, he had dysarthria and generalized action-induced choreoathetosis involving the limbs and trunk. At rest he had no involuntary movements or dystonic posturing. There was dystonic posturing of the upper limbs at with arms outstretched (Video 1 Segment 3). There was absent saccadic upgaze with normal pursuit eye movements. He had head drop and axial hypotonia causing truncal flexion. The lower limb tone was spastic and reflexes were generally brisk with extensor plantar responses. Strength and sensory examination was otherwise normal. Coordination could not be assessed due to frequency of involuntary movement disorder. He ambulated in a highly distinctive ‘frog-like’ motion; he would sit with his legs crossed, uncross them to propel his body forwards, as he was unable to maintain his trunk in an upright position due to axial hypotonia. He would use his arms to commando crawl (Video 1 Segment 3).

His investigations were summarized in Supplementary Material Table 2.

K1 Case 2

This forty-four-year-old Caucasian woman presented with episodic generalized choreoathetoid movements with dystonic posturing at age three years. She was born to non-consanguineous parents and was the product of a pregnancy complicated by pre-eclampsia at near term. Labor was complicated by breech presentation and she was born via an uncomplicated Caesarean section. There was no history of birth asphyxia or peri-natal complications. She did not crawl by the age of nine months and at age three, she developed episodic generalized choreoathetoid movements with dystonic posturing. These movements lasted for minutes to one hour and occurred in clusters as frequent as every fifteen minutes, up to seven times per day. Twice a year the clusters of similar involuntary movements lasted for twenty-four hours. The longest duration without involuntary movements was minutes. She was diagnosed with dyskinetic cerebral palsy. These movements were sufficient to arouse the suspicion of epilepsy. Trials of levodopa up to 10mg/kg daily, trihexyphenidyl, tetrabenazine or anticonvulsants or oxazepam did not help. Episodic exacerbations of involuntary movements occurred during the evening when attempting to sleep and prevented a restful sleep. Other exacerbating factors included: attempted action, emotional stress, inter-current illness and menses. There was mild improvement of the involuntary movement and sleep with clonazepam. Between episodic exacerbations, she had continuous orofacial dystonic movements and severe axial hypotonia. She could sit at age seven and walked with support as a teenager. Generalized choreoathetosis became constant, combined with axial hypotonia caused motor regression and she was unable to walk with support at age twenty-five. She required a full time carer for assistance with all activities of daily living. She has mild intellectual disability and completed ten years of education at a special school. One of her two sons is also affected (K1-1).

On examination at age thirty-one, she had dystonic dysarthria. At rest, there were continuous facial dystonic movements with blepharospasm and facial grimacing (Video 1 Segment 2), axial hypotonia with head drop and truncal flexion. There were no involuntary movements of the limbs at rest. During attempted action, she had dystonic spooning of the fingers, retrocollis and mild opisothonus with generalized choreoathetoid movements (Video 1 Segment 1 and 3). There was absent saccadic upgaze with normal pursuit eye movements. She had motor impersistence of the tongue and upper limbs. Lower limb reflexes were brisk with ankle clonus and bilateral extensor plantar responses. The motor and sensory exam was otherwise normal. Coordination could not be assessed due to frequency of involuntary movement disorder. She could not walk without assistance. Her investigations are summarized in Supplementary Material Table 2.

K2 Case 1

A thirty-two-year-old Caucasian female was born to non-consanguineous parents. Her delivery was at term and uncomplicated. She had post-natal respiratory difficulty and required an incubator for four days. She had failure to thrive, poor sucking and was a floppy baby at nine months of age. She was diagnosed with gastro-esophageal reflux at nine months. At thirteen months of age, she developed episodic generalized choreiform movements with dystonic posturing four to seven times a day. Each episode lasted five minutes to one hour. The longest absence from involuntary movements was two days. She had severe axial hypotonia, continuous orobuccal chorea with upper and lower limb dystonic posturing between episodes of involuntary movements. The episodic nature of the involuntary movements led to initial diagnosis of epilepsy. She tried phenobarbitone, sodium valproate (20mg/kg/day), carbamazepine (400mg/day) and levodopa (20mg/kg/day) without improvement. Episodic movements were exacerbated by action, sleep and intercurrent illness. These movements were severe during drowsiness and prevented her from restful sleep. There was mild reduction in sleep induced involuntary movements with clonazepam (3mg/day). She had delayed motor milestones, sitting at eighteen months and walked with assistance at two and a half years. She was diagnosed with dyskinetic cerebral palsy at twenty months of age. As a child, severe axial hypotonia prevented her from supporting her torso and her ambulation was also distinctive and ‘frog-like’ – she would sit on her crossed legs and propel her body forwards, while also using her arms to commando crawl (Video 2 Segment 1). Language was delayed, achieving the skills of a nine-month-old at age nineteen months. At age twenty-two, she still had involuntary episodes of generalized chorea with dystonic posturing with unaltered frequency (Video 2 Segment 2).. Episodic movements were exacerbated by caffeine and menstruation. However, having lost the ability to walk for a few years, she could again walk independently for 3 meters on levodopa 1000mg per day and clonazepam 4.5mg per day (Video 2 Segment 3). At age twenty-seven years, mobility and communication regressed due to increased frequency of involuntary movements. She could no longer walk independently and required assistance (Video 2 Segment 4). At age thirty-three years, three years post bilateral pallidal DBS, there was marked reduction in action-induced choreoathetosis of the limbs (Video 2 Segment 5).

Limited details were available about her mother who also had an infantile onset movement disorder, associated with mild developmental delay, dysarthria, generalized choreoathetosis and dystonic posturing of the lower limbs. Her four half-siblings were not affected.

On examination of our proband at age twenty-three years, she had intermittent generalized choreoathetoid movements precipitated by movement on a background of continuous orobuccal chorea and dystonic posturing of bilateral lower limbs. She had a slow, negative head tremor as well as rest tremor of both upper limbs and the right leg. She had motor impersistence of tongue protrusion and axial hypotonia with head drop. She had absent vertical saccades and initiated horizontal saccades with head thrust but pursuit eye movements were normal. There was lower limb spasticity with generalized hyperreflexia, ankle clonus and bilateral extensor plantar response. Sensory examination was normal. Coordination could not be assessed due to frequency of involuntary movement disorder.

At age thirty years, as episodic movements became more frequent and of longer duration for four years, she underwent bilateral globus pallidus interna deep brain stimulation (DBS). Pre-operative Burke-Fahn Marsden dystonia movement scale was seventy-five with a disability score of twenty-five points. Three years post-operative Burke-Fahn Marsden dystonia movement scale was thirty-eight with a disability score of twenty-four points. The episodic choreoathetoid and dystonic movements improved within days after surgery and she can now use a communication board more effectively and steer her electric wheelchair with a joystick. However, axial hypotonia remained unchanged and she still requires assistance for walking. During temporary cessation of DBS as part of impendence check, upper limb choreoathetosis with dystonic posturing reemerged within seconds and disappeared after reinstatement of DBS. Three years post DBS, episodic choreoathetoid movements remain suppressed during stimulation and her level of function remain unchanged. (Video 2 Segement5) Her investigations were summarized in Supplementary Material Table 2.

K3 Case 1

He is a twenty-nine-year-old Caucasian man who was born to non-consanguineous parents and the product of normal pregnancy and uneventful elective Caesarean section at term. There was no history of birth asphyxia or perinatal complications. He first presented at age fourteen months when he was not sitting and had episodic generalized choreoathetosis as well as separate episodic dystonic spasms. The frequency of the episodic choreoathetosis was three to seven times per day, lasting thirty seconds to one hour. The longest absence from involuntary movements was four hours. Both choreoathetoid and dystonic movements were precipitated by action, alcohol and severely worsened during drowsiness, precluding him from restful sleep. Only the dystonic movements worsened with laughter or sneezing. The episodic movements was initially diagnosed and treated as epilepsy. Levodopa (300mg daily for twelve months), clonazepam 0.25mg daily, trihexyphenidyl, pregabalin (25mg daily), gabapentin, baclofen and carbamazepine 200mg daily were ineffective. Clobazam 15mg per day reduced nocturnal choreoathetosis and enabled more restful sleep. He had axial hypotonia without dystonic posturing or spasticity between episodes of involuntary movements. He never achieved independent ambulation and at age three years, walked with assistance of a walker with a supportive belt due to axial hypotonia. There was no motor regression. Language milestones were normal and intellectual ability was preserved. His dominant hand had no functional use but he can still use the non-dominant hand to operate an electric wheelchair and computer mouse. His parents and two sisters are unaffected.

On examination at age twenty-three years, he had dysarthria and no involuntary movement at rest. When attempting to move his arm or legs, he had action-induced generalized choreoathetoid movements of the upper and lower limbs (Video 3 Segment 1). Laughter precipitated bilateral dystonic posturing of the arms, extension of legs and extension of the neck. He had absent voluntary saccadic upward gaze and long horizontal and downward saccadic latency. Pursuit eye movements were normal apart from mild upgaze restriction. There was motor impersistence of tongue and finger grip. The motor, reflex and sensory examination was otherwise normal. Coordination could not be assessed due to frequency of involuntary movement disorder. He needed the assistance of two people to stand and walked a few steps due to axial hypotonia and exacerbation of generalized choreoathetosis. His investigations were summarized in Supplementary Material Table 2.

K4 Case 1

A nine-year-old Caucasian girl was born to non-consanguineous parents of Italian ancestry. She was the product of a normal pregnancy and elective uncomplicated Caesarean section without postnatal complications. She was a ‘floppy baby’ at age five months. There was motor milestone delay with hypotonia and inability to sit at six months. She had generalized chorea between the age of six to twelve months. She managed to sit unsupported for thirty seconds at age five and has not yet managed to crawl or walk independently. She walked with support of a walker or her parent from age five and there was no motor regression. She had initial language milestone delay, could speak three words at age two and a half but can now converse in full sentences using two languages. She had dysphagia from age three to five and had recurrent aspiration pneumonia. She had normal intelligence and attends mainstream school. At rest, she had generalized choreiform movements, which were exacerbated in severity and duration by attempted movement, mental concentration, early morning sleep, high ambient or body temperature. She could sleep for four hours without exacerbation of choreiform movements, which disturbed sleep after midnight. In addition, every two to three weeks, she had involuntary movements that were not associated with choreiform movements. These consisted of generalized dystonic spasms with head turning, mouth grimacing and limb stiffening, associated with anarthria and fatigue. These occurred in clusters for two to three days and lasted several minutes each episode, without associated triggers. Clobazam (10mg/day) or carbamazepine reduced choreiform movements and provided more restful sleep. She could feed herself but required assistance for writing, driving the wheelchair and other activities of daily living. Her parents and two half siblings are unaffected.

On examination at age nine years she had generalized chorea and dysarthria. Vertical upward saccadic latency was prolonged but normal in velocity and range. There was normal horizontal saccadic velocity and range. Pursuit eye movements were smooth. Tone was normal in the limb and reduced in the neck and trunk. Strength was normal and there was motor impersistence of the tongue and limb. Reflexes were normal and symmetrical with bilateral flexor plantar responses. Sensory examination was normal. Coordination could not be assessed due to frequency of involuntary movement disorder. She could not roll over on the floor and required assistance to stand and walk. Her arm had intermittent dystonic posturing when walking. Her investigations were summarized in Supplementary Material Table 2.

K5 Case 1

A twenty-nine-year-old Caucasian male was born to non-consanguineous parents after an uneventful pregnancy. He was born six weeks premature after an uneventful delivery. He had severe gastric reflux at nine months age and underwent Nissen fundoplication surgery at age four. There was motor and language milestone delay but he had normal intelligence. He walked at age three years. Generalized involuntary movement started at six months of age. Over several years he had progression of involuntary movements resulting in speech and gait disturbance. These were episodic myoclonic and dystonic spasms lasting ten seconds to two minutes with painful trunk hyperextension, neck and legs, leading to falls or slipping out of his seat. Involuntary movements occurred daily and the longest remission was hours. These were worse during sleep and caused insomnia and generalized body pain. A levodopa trial of 600mg/day was unsuccessful but clonazepam 4mg daily helped reduce episodic involuntary movements and subsequent falls. After clonazepam, he could sleep better, dress himself, hold a cup with one hand, use cutlery and a computer keyboard.

At age nine years he was diagnosed with cerebral palsy and had behavioral problems with aggression. From age fourteen years he was on risperidone 0.5mg twice a day. At age fourteen he had left hip dislocation.

Limited history is available about his mother and maternal grandfather who were also affected with mild involuntary movements, speech and balance problems. His grandfather’s involuntary movements were worse in the morning and improved with caffeine. His mother was similarly affected with motor and language milestone delays and childhood onset involuntary movements without progression (Video 3 Segment 2). The grandfather who was enrolled in a brain donor program died at age eighty-four and his brain had widespread Lewy body pathology in the rhinal cortices, amygdala and substantia nigra, in addition cerebellar molecular layer neurons had abnormal nuclear envelopes and loss of nuclear calcium staining with peri-nuclear ring aggregations^1^. The findings were atypical and not consistent with Parkinson’s disease.

On examination at age sixteen years without clonazepam (Video 3 Segment 2), he had constant jerky movements of the orobuccal region, which were originally diagnosed as myoclonus. There was associated dysarthria. Saccadic and pursuit eye movements were normal. He had right torticollis, retrocollis and truncal dystonia with right lateral lean. There was associated axial hypotonia and motor impersistence of the tongue. In addition, he had myoclonic jerks (but in retrospect also compatible with a choreiform component) affecting the neck, shoulders and hip flexion. He had normal strength, reflexes and sensory examination. Plantar response was flexor. His coordination and finger tapping was performed slowly but accurately. Examination at age twenty-seven with clonazepam 4mg per day, there was reduction in generalized myoclonic movements. His investigations were summarized in Supplementary Material Table 2.

K6 Case 1

A nine-year-old Caucasian girl, was born to non-consanguineous parents after an uneventful pregnancy, complicated by emergency Caesarean section for failure to progress. She was admitted to the Special Care Baby Unit for irritability, but discharged after twenty-four hours. The early neonatal course was uneventful.

She had normal motor and language milestone. Abnormal movements were noticed from six months of age. Initially, she was noted to have episodic generalized dyskinetic movements involving face, hands, feet, shoulders and hips. These lasts a few seconds, and occurring one to twenty times every day, sometimes in clusters, with exacerbations lasting several weeks. Movements occurred at any time of the day, and in a number of situations (playing, sitting, feeding) but not during sleep early in the disease course. In order to treat her dyskinetic symptoms a number of medications were trialled, including carbamazepine (age three years), acetazolamide (age three to four years), sodium valproate (age four to seven years) and levetiracetam (age seven years). Carbamazepine led to worsening of the motor disorder. Acetazolamide led to transient improvement of speech, gait and fine motor skills, but this effect was not sustained. On sodium valproate therapy, she showed mild improvement of dyskinesia, and movements worsened if a dose was omitted. She remained asymptomatic in between these episodes. She is unsteady on standing and may fall but did not have motor regression. There was a family history of multiple-system atrophy (MSA) and progressive late onset spino-cerebellar ataxia in her grandfather, who died age sixty. Both parents were healthy and appeared unaffected.

On review with a paediatric neurologist at the age of two and a half years, neurological examination was normal.

From the age of three to five years, abnormal movements worsened and speech started to deteriorate. Initially, the episodic movements increased in frequency and occurred daily. Movements appeared on waking, were more prominent in the mornings and improved over the course of the day. At night involuntary movements disrupted her sleep pattern. She manifested repetitive blinking and orolingual dyskinesia. There was evidence of limb dyskinesia, as well as truncal and gait instability, with inturning of her right lower leg and frequent falls. Her speech became slurred. Over the last four years, the movement disorder has stabilized but is now no longer episodic, but a constant clinical feature. On examination she had evidence of generalized chorea, involving her head, neck and limbs with a predominantly distal pattern (Video 3 Segment 4). There was also a head tilt to the left.

Reference

1. McCann H, Fung VS, Klein C, Halliday GM. Unusual alpha-synuclein and cerebellar pathologies in a case of hereditary myoclonus-dystonia without SGCE mutation. Neuropathology and applied neurobiology. 2015 Jan 13.
