# Supplementary material for: Phenotypic insights into ADCY5‐associated disease
Source: Mov Disord. 2016 Apr 8;31(7):1033–40. doi: 10.1002/mds.26598 (PMC4950003; doi:10.1002/mds.26598)
Supplement: Supplementary file 2 — Supplementary Information 2 [file MDS-31-1033-s002.docx]

Supplementary Material

**METHOD**

Whole exome sequencing

The capture was done with Agilent SureSelect Target Enrichment V5 (Agilent Technologies, Santa Clara, CA, USA) pull-down array. The Illumina HiSeq 2000 platform (Illumina, Inc. San Diego, CA) was used to perform whole exome next-generation sequencing. Reads were aligned to the reference genome (GRCh37/hg19) using the Burrows-Wheeler Alignment tool and single nucleotide variants (SNVs) and small insertion/deletions (Indels) were identified by using SAMtools^1, 2^. For each sample, variant sites (SNVs and Indels) were called using the GATK Unified Genotyper^3^. The calls were then annotated with vcf-annotate^4^. Functional annotations were added using the Ensembl Variant Effect Predictor v73 (VEP) against Ensembl 73^5^. VEP v73 also was used to provide coding consequence predictions, SIFT, Polyphen, GERP conservation and Condel scores^6-9^. The annotation and the corresponding protein consequence performed with VEP v73 reflects the most severe impact amongst the gene transcripts, which may not necessarily correspond to the GenBank mRNA NCBI Reference Sequence in all of the cases. Subsequently, standard sequence quality control criteria were applied to the called variants: variants with phred-scaled quality score (QUAL)> 90 and mapping quality (MQ)> 45 were investigated further. In addition, only rare variants with a minor allele frequency of <1% in all of the following datasets were considered for downstream analyses: 1000 genomes, UK10K twins cohort and NHLBI Exome Sequencing Project^10^. Furthermore, only putative functional (i.e. non-synonymous, stop codon loss or inframe deletions) and loss of function variants were analysed (i.e. nonsense, frameshift and essential splice site variants).

Reference

1. Li H, Durbin R. Fast and accurate short read alignment with Burrows-Wheeler transform. Bioinformatics. 2009 Jul 15;25(14):1754-60.

2. Li H, Handsaker B, Wysoker A, et al. The Sequence Alignment/Map format and SAMtools. Bioinformatics. 2009 Aug 15;25(16):2078-9.

3. McKenna A, Hanna M, Banks E, et al. The Genome Analysis Toolkit: a MapReduce framework for analyzing next-generation DNA sequencing data. Genome research. 2010 Sep;20(9):1297-303.

4. Danecek P, Auton A, Abecasis G, et al. The variant call format and VCFtools. Bioinformatics. 2011 Aug 1;27(15):2156-8.

5. McLaren W, Pritchard B, Rios D, Chen Y, Flicek P, Cunningham F. Deriving the consequences of genomic variants with the Ensembl API and SNP Effect Predictor. Bioinformatics. 2010 Aug 15;26(16):2069-70.

6. Cooper GM, Stone EA, Asimenos G, et al. Distribution and intensity of constraint in mammalian genomic sequence. Genome research. 2005 Jul;15(7):901-13.

7. Kumar P, Henikoff S, Ng PC. Predicting the effects of coding non-synonymous variants on protein function using the SIFT algorithm. Nature protocols. 2009;4(7):1073-81.

8. Adzhubei IA, Schmidt S, Peshkin L, et al. A method and server for predicting damaging missense mutations. Nature methods. 2010 Apr;7(4):248-9.

9. Gonzalez-Perez A, Lopez-Bigas N. Improving the assessment of the outcome of nonsynonymous SNVs with a consensus deleteriousness score, Condel. Am J Hum Genet. 2011 Apr 8;88(4):440-9.

10. NHLBI GO Exome Sequencing Project (ESP). Seattle, WA Available from: http://evs.gs.washington.edu/EVS/.
