# Supplementary figures and images for: Phenotypic insights into ADCY5‐associated disease
Source: Mov Disord. 2016 Apr 8;31(7):1033–40. doi: 10.1002/mds.26598 (PMC4950003; doi:10.1002/mds.26598)

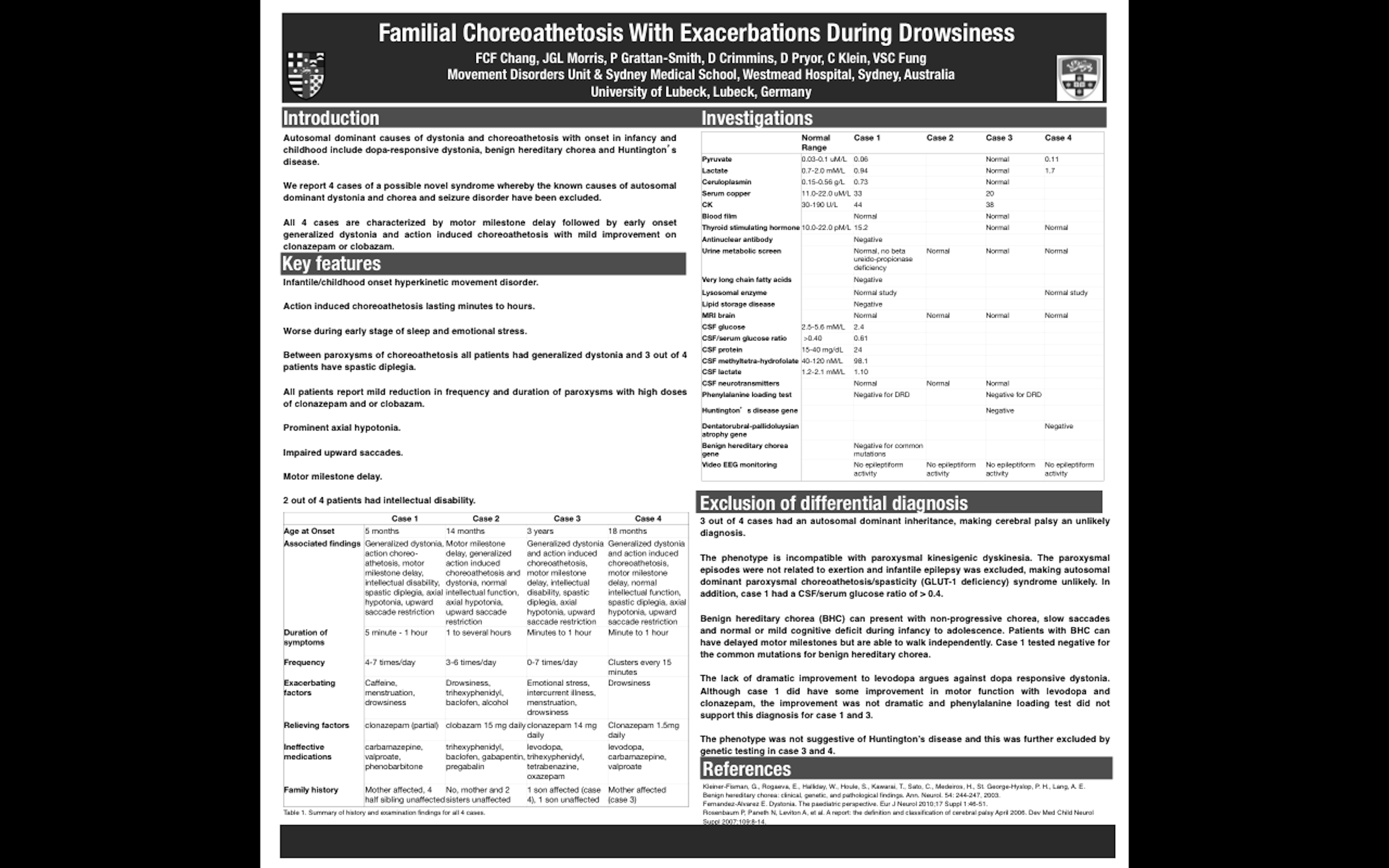

Supplement: Supplementary file 3 — Supplementary Information Figure 1 [file MDS-31-1033-s003.tif]

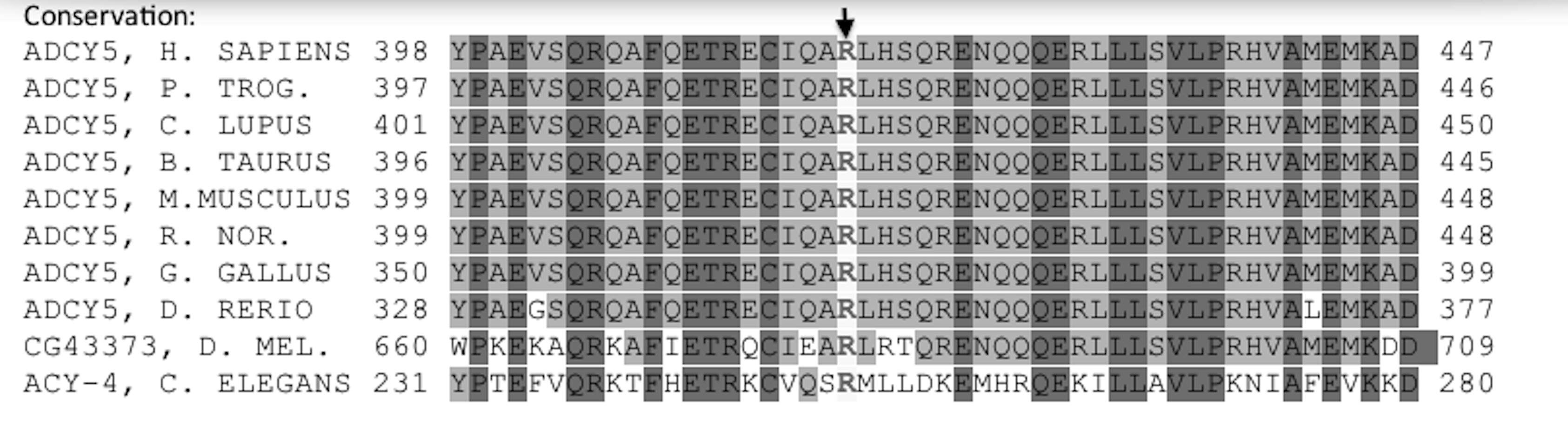

Supplement: Supplementary file 4 — Supplementary Information Figure 2 [file MDS-31-1033-s004.tif]

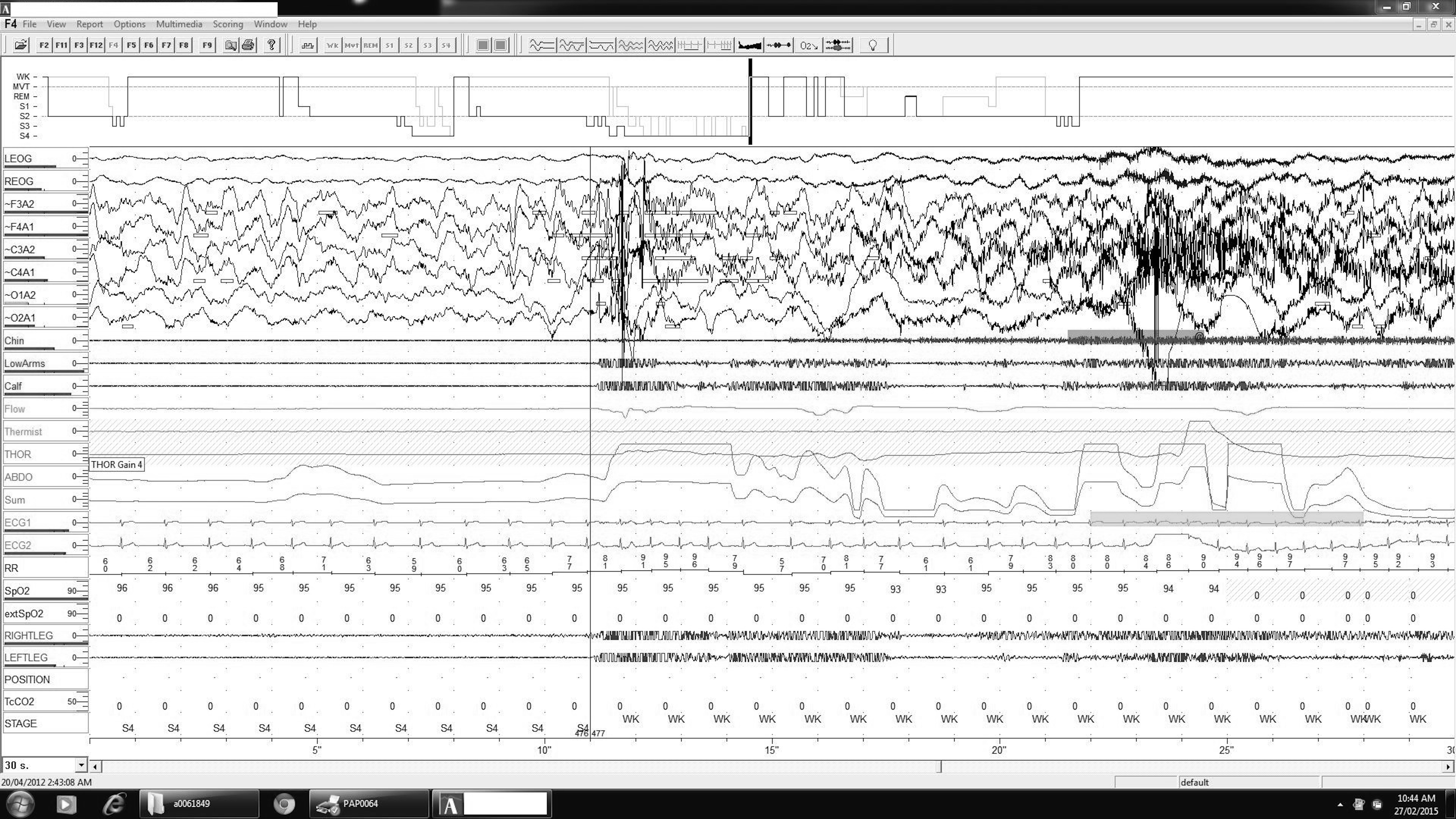

Supplement: Supplementary file 5 — Supplementary Information Figure 3 [file MDS-31-1033-s005.JPG]

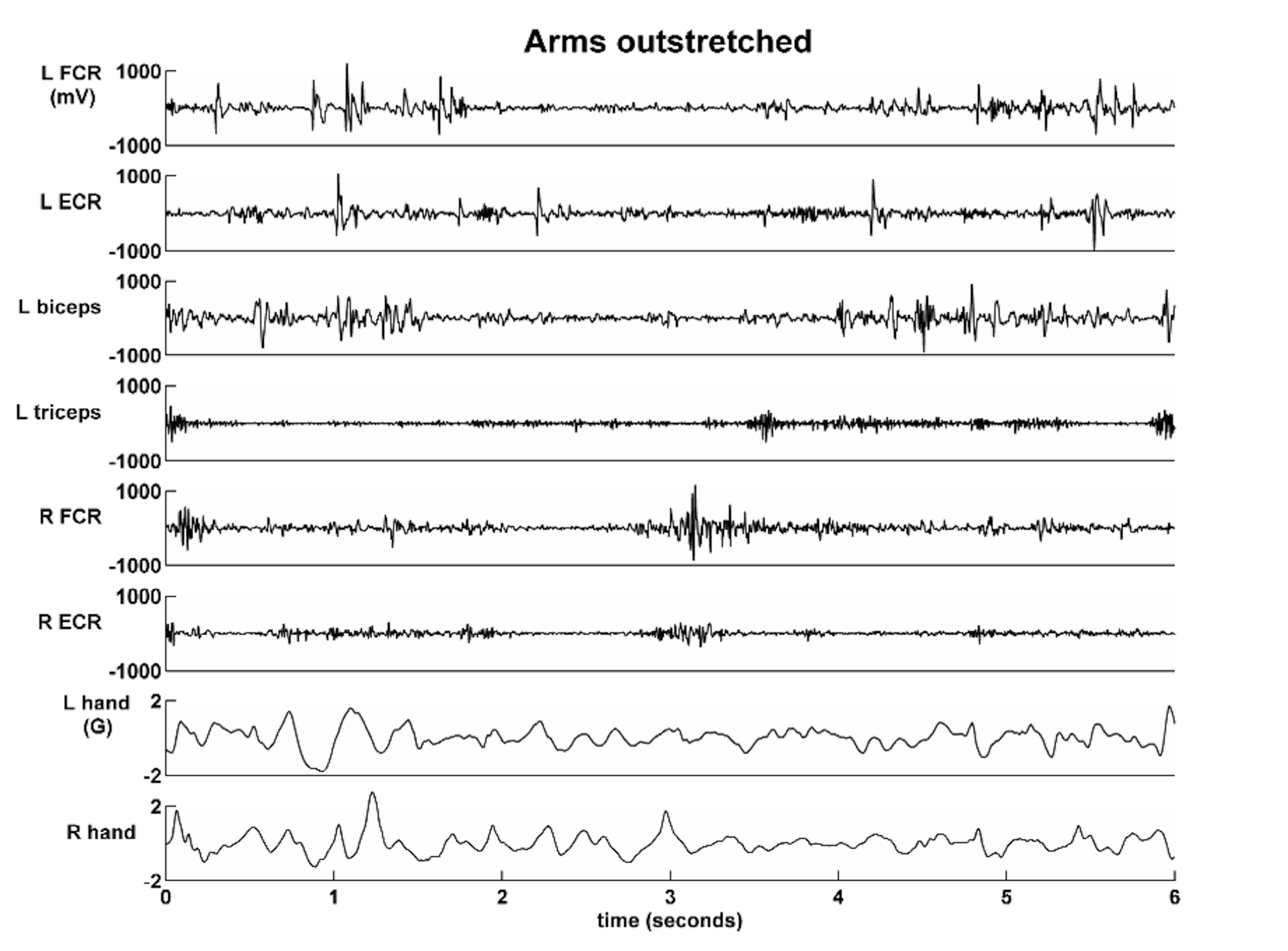

Supplement: Supplementary file 6 — Supplementary Information Figure 4 [file MDS-31-1033-s006.tif]

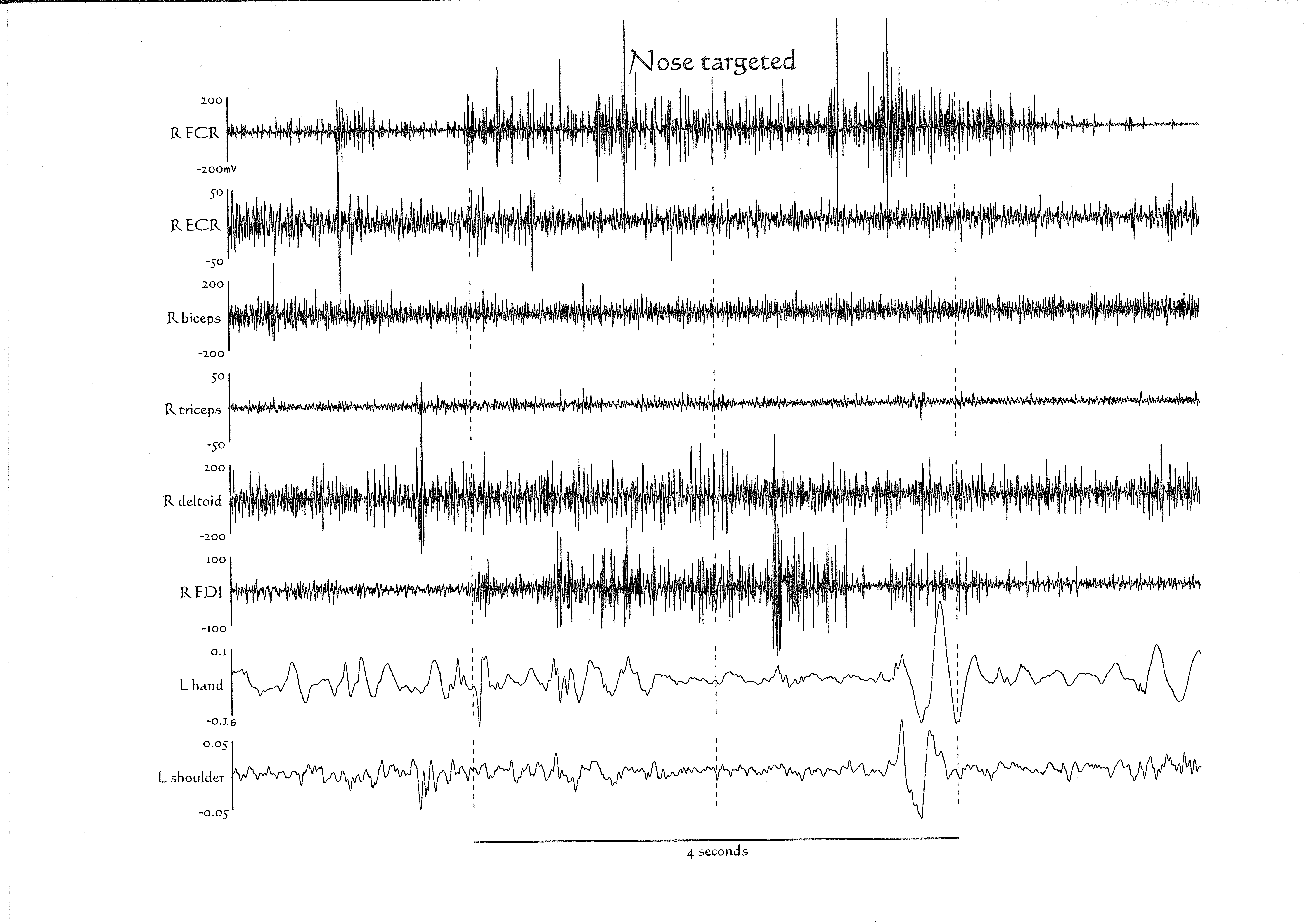

Supplement: Supplementary file 7 — Supplementary Information Figure 5 [file MDS-31-1033-s007.tif]
