# Supplementary material for: Phenotypic insights into ADCY5‐associated disease
Source: Mov Disord. 2016 Apr 8;31(7):1033–40. doi: 10.1002/mds.26598 (PMC4950003; doi:10.1002/mds.26598)
Supplement: Supplementary file 8 — Supplementary Information Table 1 [file MDS-31-1033-s008.docx]

| **Kindred(number)-Case** | **K1-1 #** | **K1-2 #** | **K2-1 #** | **K3-1 #** | **K4-1** | **K5-1 *#** | **K6-1 #** | **K7-1** | **K8-1** | **K9-1** | **K9-2** |
| --- | --- | --- | --- | --- | --- | --- | --- | --- | --- | --- | --- |
| Reference | Our paper | Our paper | Our paper | Our paper | Our paper | Our paper | Our paper | Chen et al 2014 | Chen et al 2014 | Fernandez 2001, Chen 2012 | Fernandez 2001, Chen 2012 |
| Gene mutation | c.1252C>T | Not tested, mother of K1-1 | c.1252C>T | c.1252C>T | c.1252C>T | c.1252C>G | c.1253G>A | c.1252C>T | c.1252C>T | c.2176G>A | c.2176G>A |
| Protein | p.R418W | - | p.R418W | p.R418W | p.R418W | p.R418G | p.R418Q | p.R418W | p.R418W | p.A726T | p.A726T |
| Age onset (years) | 1 | 3 | 1 | 1 | 0.5 | 0.5 | 0.5 | 1.3 | 5 | adolescence | adolescence |
| Dystonia | Episodic generalized | Episodic generalized | Episodic generalized | Episodic generalized | Episodic generalized | Episodic generalized, lasting second-2 minutes | No | Paryoxysmal generalized | Generalized | NM | MN |
| Choreoathetosis | Generalized | Generalized | Generalized | Generalized | Generalized | - | Generalized | Paroxysmal generalized | Paroxysmal generalized | arm and face | paroxysmal then constant |
| Episodic exacerbations of choreoathetosis | minutes | minutes to 1 hour | 5 minutes to 1 hour | 30 seconds to 1 hour | minutes | - | Constant | NM | hours to days | NM | NM |
| Frequency | clusters up to every 15 minutes | 0-7 times a day | 4 to 7 times a day | 3-7 times a day | Daily | - | Constant | Several times per day | Daily | NM | NM |
| Exacerbating factors | Action, drowsiness, intercurrent illness, emotional stress | Action, drowsiness, intercurrent illness, emotional stress | Action, drowsiness, emotional stress, caffeine | Action, drowsiness, stress | Action, mental concentration, early morning sleep, high ambient or body temperature |  | Action, drowsiness | Anxiety, stage N2 and N3 sleep | NM | stress | NM |
| Medication trial | Levodopa, carbamazepine, valproate | Levodopa, trihexyphenidyl, tetrabenazine, oxazepam | Phenobarbitone, valproate, carbamazepine, levodopa | Levodopa, clonazepam, trihexyphenidyl, baclofen, carbamazepine | Clonazepam, carbamazepine | Levodopa | Carbamazepine, acetazolamide, levetiracetam, sodium valproate |  | NM | NM | deanol, primidone,chlordiazepoxide,amitriptyline, trifluoperazine,acetazolamide |
| Improvement | Clonazepam 0.5mg | clonazepam 14mg daily | clonazepam 4.5mg | Clobazem 15mg daily | Clobazem 10mg/day, cabamazepine | Clonazepam 4mg, caffeine | None | NM | With activity | NM | - |
| Facial dyskinesia | + | + | + | - | - | + | + | + | + | - | + |
| Axial hypotonia | + | + | + | + | + | + | - | + | - | NM | NM |
| Myoclonus | - | - | - | - | - | Multifocal | Upper limb | All limbs | NM | NM | NM |
| Spasticity | + | + | + | - | - | - | - | + | - | - | - |
| Intellectual disability | - | + | + | - | - | - | - | NM | NM | - | - |
| upward gaze palsy | + | + | + | + | - | - | - | NM | NM | NM | NM |
| Motor regression | - | + | + | - | - | - | - | + | - | - | - |
| Epilepsy | - | - | - | - | - | - | - | - | - | - | - |
| Ethnicity | Australian-Caucasian | Australian-Caucasian | Australian-Caucasian | Australian-Caucasian | Italian | Australian-Caucasian | Australian-Caucasian | European | European | German | German |

| **K9-3** | **K9-4** | **K9-5** | **K9-6** | **K10-1** | **K10-2** | **K11-1** | **K11-2** | **K12-1** | **Total no. cases (%)** |
| --- | --- | --- | --- | --- | --- | --- | --- | --- | --- |
| Fernandez 2001, Chen 2012 | Fernandez 2001, Chen 2012 | Fernandez 2001, Chen 2012 | Fernandez 2001, Chen 2012 | Carapito 2014 | Carapito 2014 | Mencacci 2015 | Mencacci 2015 | Mencacci 2015 |  |
| c.2176G>A | c.2176G>A | c.2176G>A | c.2176G>A | c.2088+1G>A | c.2088+1G>A | c.1252C>T | c.1252C>T | c.1252C>T |  |
| p.A726T | p.A726T | p.A726T | p.A726T | haploinsufficiency | haploinsufficiency | p.R418W | p.R418W | p.R418W |  |
| NM | 5 | 6 | early childhood | 0.5 | 4 | 1 | 1 | 2 |  |
| NM | paroxysmal limbs | paroxysmal limbs | No | Limbs and neck | Hands | No | Generalized | Generalized | 14 (70%) |
| NM | paroxysmal limbs and face | paroxysmal limbs and face | Paroxysmal arm and neck then constant | Face, toes and fingers | Limbs, trunk, face | Generalized | Generalized | Generalized | 19 (95%) |
| NM | 2 minute | 2 minute | NM | NM | NM | NM | NM | NM |  |
| NM | daily | daily | NM | NM | NM | NM | NM | NM |  |
| stress | stress, excitement | stress, excitement | stress, excitement | NM | NM | Awakening | Action, awakening | Action, awakening, stress, anxiety |  |
| propranolol | carbamazepine, valproic acid | carbamazepine, valproic acid | NM | NM | NM | Tetrabenazine | Tetrabenazine, baclofen, trihexyphenidyl, baclofen, levodopa, clonazepam | NM |  |
| Yes, propranolol | Yes acetazolamide | Yes acetazolamide | NM | NM | NM | Yes trihexyphenidyl | Yes trihexyphenidyl and tetrabenazine | Yes trihexyphenidyl and tetrabenazine |  |
| + | - | - | + | - | - | - | - | + | 11 (55%) |
| NM | - | - | NM | - | + | - | - | - | 8 (40%) |
| NM | NM | NM | NM | NM | NM | No | No | No | 2 (10%) |
| - | - | - | - | + | + | - | - | - | 6 (30%) |
| - | - | - | - | - | - | - | - | - | 2 (10%) |
| NM | - | - | NM | - | - | + | + | + | 7 (35%) |
| - | - | - | - | + | + | - | - | + | 7 (35%) |
| - | + | - | NM | - | - | - | - | - | 1 (5%) |
| German | German | German | German | French | French | UK-Caucasian | UK-Caucasian | Pakistani |  |

Supplementary Material Table 1. Summary of *ADCY5* mutation clinical presentation, phenomenology and genotypes reported to date.

*- histopathology of affected materal grandfather revealed Lewy Body pathology; #- video available; NM- not mentioned;
