# Supplementary material for: Phenotypic insights into ADCY5‐associated disease
Source: Mov Disord. 2016 Apr 8;31(7):1033–40. doi: 10.1002/mds.26598 (PMC4950003; doi:10.1002/mds.26598)
Supplement: Supplementary file 9 — Supplementary Information Table 2 [file MDS-31-1033-s009.docx]

Table 2. Summary of investigations

| Family-subject number | K1-1 | K1-2 | K2-1 | K3-1 | K4-1 | K5-1 | K6-1 |
| --- | --- | --- | --- | --- | --- | --- | --- |
| Serum pyruvate | N | N | N |  |  |  |  |
| Serum lactate | N | N | N |  |  |  | N |
| Caeruloplasmin |  | N | N |  | N | N |  |
| Serum copper |  | N | N |  | N | N |  |
| Creatine Kinase |  | N | N |  | N | N |  |
| Blood film |  | N | N |  |  | N |  |
| Thyroid function | N | N | N |  | N | N | N |
| Vitamin B 12 |  |  | N |  |  | N |  |
| Antinuclear antibody |  |  | Neg |  |  | N |  |
| Extractible nuclear antigen |  |  | Neg |  |  | N |  |
| Hepatitis B serology |  |  | Neg |  |  |  |  |
| MRI brain | N | N | N | N | N | N | N apart from low cerebellar tonsil |
| Very long chain fatty acids |  |  | N |  | N |  |  |
| Lysosomal enzyme | N |  | N |  | N |  |  |
| Lipid storage disease |  |  | N |  |  |  |  |
| Urine metabolic screen | N | N | N | N | N |  |  |
| Electroencephalography and video telemetry | N | N | N | N | N | N | N |
| Surface electromyography |  |  |  | action induced choreoathetosis |  | Myoclonus and dystonic EMG activity |  |
| Neuropsychological testing |  | Frontal impairment | Low average | N |  |  |  |
| CSF glucose |  | N | N |  | N |  |  |
| CSF/serum glucose ratio |  |  | N |  | N |  |  |
| CSF protein |  | N | N |  | N |  |  |
| CSF methyl-tetrahydrofolate |  |  | N |  |  |  |  |
| CSF lactate |  |  | N |  | N |  |  |
| CSF neurotransmitters |  | N | N | N | N |  |  |
| Huntington disease triplet expansion |  | Neg |  |  |  |  |  |
| *DYT1* gene mutation |  |  |  |  |  | Neg |  |
| *SGCE* gene mutation |  |  |  |  |  | Neg | Neg |
| *DRPLA* gene mutation | Neg |  |  |  |  |  |  |
| Benign hereditary chorea (*NKX2-1* gene mutation) |  |  | Neg |  | Neg |  | Neg |
| *POLG* mutation |  |  |  |  | Neg |  |  |
| Fragile X mutation |  |  |  |  | Neg |  |  |
| *SLC2A1* mutation |  |  |  |  |  |  | Neg |
| Other genetic testing ^#^ |  |  |  |  |  |  | Neg |
| Common mitochondrial mutations* |  |  |  |  |  |  | Neg |
| Karyotype |  |  |  |  | N |  |  |

N- normal; Neg-negative; *DYT1*- torsion dystonia 1; *DRPLA*- dentatorubral-pallidoluysian atrophy; *NKX2-1* –thyroid transcription factor 1; *POLG*- Polymerase gamma.

*- includes: *NARP* 8993G and C, *MELAS* 3243, *MERRF* 8344 mutations.

*#- ATP1A3, MICU1, GCH1, SR, DHPR, DDC, TH, SLC6A3, VMAT2* mutations.
