# Supplementary material for: Phenotypic insights into ADCY5‐associated disease
Source: Mov Disord. 2016 Apr 8;31(7):1033–40. doi: 10.1002/mds.26598 (PMC4950003; doi:10.1002/mds.26598)
Supplement: Supplementary file 10 — Supplementary Information Table 3 [file MDS-31-1033-s010.docx]

Supplementary Material Table 3

*ADCY5* variants affecting arginine at the position 418 and their predicted pathogenicity and CADD scores.

| *ADCY5* Change | MutationTaster | PolyPhen2 | SIFT | CADD |
| --- | --- | --- | --- | --- |
| c.1252C>T (p.R418W) | Disease causing | Probably damaging | Damaging | 34 |
| c.1252C>G (p.R418G) | Disease causing | Probably damaging | Damaging | 29.1 |
| c.1253G>A (p.R418Q) | Disease causing | Probably damaging | Damaging | 35.0 |
